# Aquaporin Expression Contributes to Human Transurothelial Permeability In Vitro and Is Modulated by NaCl

**DOI:** 10.1371/journal.pone.0045339

**Published:** 2012-09-24

**Authors:** Peter C. Rubenwolf, Nikolaos T. Georgopoulos, Lisa A. Kirkwood, Simon C. Baker, Jennifer Southgate

**Affiliations:** 1 Jack Birch Unit for Molecular Carcinogenesis, Department of Biology, University of York, York, United Kingdom; 2 Department of Chemical and Biological Sciences, University of Huddersfield, Huddersfield, United Kingdom; Chinese University of Hong Kong, Hong Kong

## Abstract

It is generally considered that the bladder is impervious and stores urine in unmodified form on account of the barrier imposed by the highly-specialised uro-epithelial lining. However, recent evidence, including demonstration of aquaporin (AQP) expression by human urothelium, suggests that urothelium may be able to modify urine content. Here we have we applied functional assays to an in vitro-differentiated normal human urothelial cell culture system and examined both whether AQP expression was responsive to changes in osmolality, and the effects of blocking AQP channels on water and urea transport. AQP3 expression was up-regulated by increased osmolality, but only in response to NaCl. A small but similar effect was seen with AQP9, but not AQP4 or AQP7. Differentiated urothelium revealed significant barrier function (mean TER 3862 Ω.cm^2^), with mean diffusive water and urea permeability coefficients of 6.33×10^−5^ and 2.45×10^−5^ cm/s, respectively. AQP blockade with mercuric chloride resulted in decreased water and urea flux. The diffusive permeability of urothelial cell sheets remained constant following conditioning in hyperosmotic NaCl, but there was a significant increase in water and urea flux across an osmotic gradient. Taken collectively with evidence emerging from studies in other species, our results support an active role for human urothelium in sensing and responding to hypertonic salt concentrations through alterations in AQP protein expression, with AQP channels providing a mechanism for modifying urine composition. These observations challenge the traditional concept of an impermeable bladder epithelium and suggest that the urothelium may play a modulatory role in water and salt homeostasis.

## Introduction

As a result of its unique structural and functional properties, the urothelium forms a urine-blood barrier, the main physiological significance of which is to minimise reabsorption of noxious excreted compounds by maintaining the composition and final concentration of the urine produced by the kidneys. Hence, the urothelial barrier is recognised as being critical to normal bladder function and metabolic homeostasis [1]. Although isotope studies have demonstrated reabsorption of ^14^C urea and ^3^H water across the bladder wall in several animal species [2], [3], [4], the potential mechanisms have not been investigated. Our group was the first to provide molecular evidence that human urothelium expresses aquaporins (AQP) 3, 4, 7 and 9, thus indicating a potential molecular basis for water and urea transport across the urothelial layer [5]. Modification of urine composition in the urinary tract [6], [7] and upregulated expression of AQP proteins in dehydrated rats [8] also challenge the traditional concept of the urinary tract as a poorly permeable transit/storage unit. Together, these observations support a more controversial hypothesis: that the urothelium is able to modify the composition and volume of urine in the lower urinary tract in response to the body's hydration status.

We have established an in vitro culture system whereby normal human urothelium isolated from surgical specimens is first expanded in vitro as normal human urothelial (NHU) cell monocultures and subsequently differentiated to form a functional, biomimetic barrier urothelium [9]. The human urothelial tissue equivalent comprises a stratified urothelium consisting of basal, intermediate and superficial cells, with differential expression of cytokeratins, superficial tight junctions, uroplakins and AQPs 3, 4 and 7 [5]. Functionally, the neotissues show many characteristics of native urothelium, including high TER of >2500 Ω.cm^2^, apical membrane-restricted amiloride-sensitive sodium ion channels, basal expression of Na^+^/K^+^-ATPase, and low diffusive permeability to urea, water and dextran [9].

Given the concordance in AQP expression between native and in vitro-generated urothelial constructs [5], we have used the latter to examine the effects of altered osmolality on AQP expression and to assess transurothelial water and urea transport in functional assays. Here we present evidence that AQP expression is highly modulated by exposure to salt and that functionally, AQP channels contribute to a low but finite flux of water and urea across urothelial tissue constructs.

## Results

### Effects of osmolality on AQP expression

NHU cells maintained on non-permeable tissue culture substrates in either proliferative or differentiated states, tolerated culture osmolalities ranging from 215 to 500 mosm/kg for at least 96 hours without morphological change, as monitored by phase contrast microscopy. Above 500 mosm/kg, cultures displayed severe morphological changes indicative of cell death (not shown). By contrast, differentiated urothelial constructs established on permeable Snapwell™ membranes exhibited increased tolerance to hyperosmotic medium (595 mosm/kg) presented to the apical surface.

**Figure 1:**
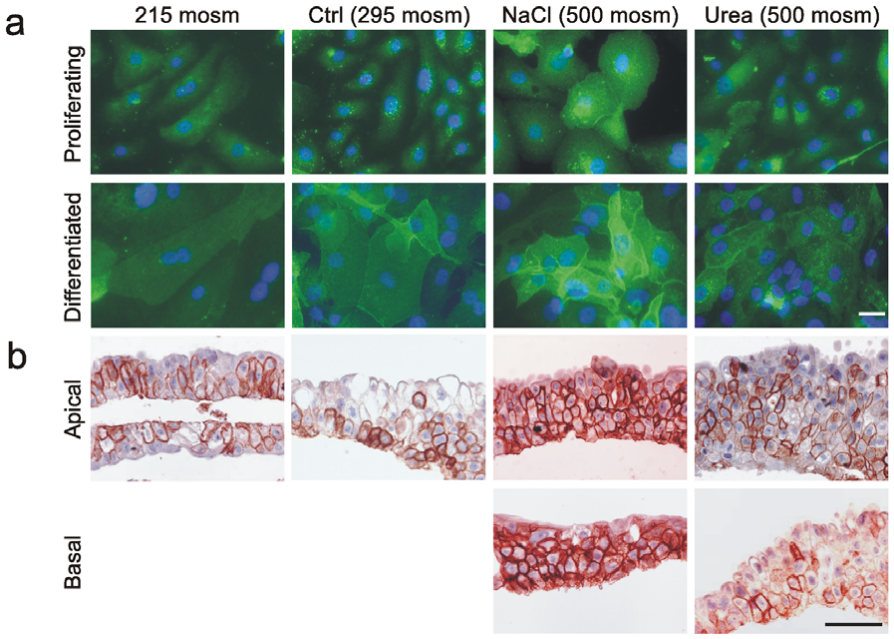
Immunolocalisation and expression of AQP3 in response to altered medium osmolality. a) Immunofluorescence labelling of non-differentiated (proliferative) and differentiated NHU cell cultures grown on glass slides showing increased labelling intensity and membrane localisation of AQP3 at high concentrations of NaCl, but not urea, following 72 hours of exposure. Scale bar: 10 µM. b) Immunohistochemistry of differentiated NHU cell constructs grown on permeable Snapwell membranes and exposed for 72 hours to indicated osmolalities. Note no effect of urea, but major increase in AQP3 expression in all layers in response to medium made hyperosmotic (500 mosm/kg) by the addition of NaCl, irrespective of exposure via apical or basal aspect. Scale bar: 50 µM.

Immunofluorescence microscopy of proliferative and differentiated NHU cell cultures revealed increased expression of AQP3 protein following exposure to NaCl-supplemented medium (500 mosm/kg), compared to cultures maintained under physiological conditions (295 mosm/kg) (Figure 1a). There was also relocalisation of AQP3 to intercellular borders in hyperosmotic medium, which was particularly apparent in differentiated cell cultures. By contrast, AQP3 immunolabelling was less intense in cultures exposed to hypo-osmotic culture (215 mosm/kg). Little change in AQP3 expression and localisation was observed in cultures exposed to hyperosmotic medium supplemented with urea (Figure 1a). These results were confirmed in sections of differentiated NHU cell culture constructs assessed by immunohistochemistry, where expression ranged from predominantly basal (290 mosm/kg) to intense expression in all layers (500 mosm/kg), irrespective of whether the apical or the basal aspect was exposed to the hyperosmotic medium (Figure 1b).

**Figure 2:**
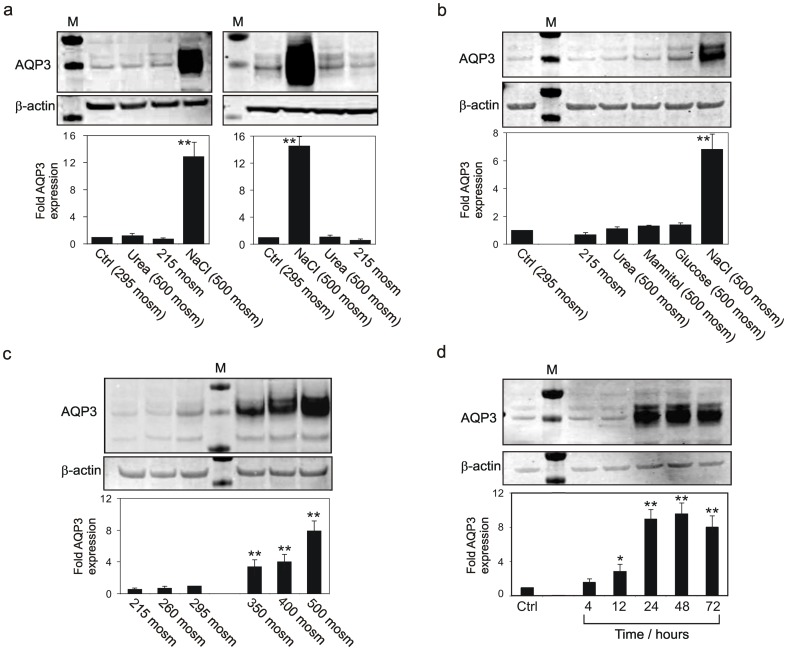
Analysis of AQP3 expression by immunoblotting. a) AQP3 expression by non-differentiated (left panel) and differentiated (right panel) NHU cell cultures following osmotic stress, compared to β-actin (loading control). Immunoblotting analysis revealed an up-regulation of AQP3 protein expression by NHU cell cultures treated for 72 hours with NaCl-supplemented hyper-osmotic medium. In hypo-osmotic culture, expression was reduced by 30% and 38%, respectively, relative to control. Urea had no effect on AQP3 protein expression. b) Exposure of non-differentiated NHU cell cultures to a range of osmotic solutes for 48 hours. NaCl induced a 6.4-fold increase in AQP3 protein expression, but other solutes had minimal effect and hypo-osmotic culture medium reduced expression. c) 48 hour responses of non-differentiated NHU cells to a range of osmotic salt concentrations. d) Time-dependency of non-differentiated NHU cell response to hyperosmotic salt. Each panel shows a representative immunoblot for AQP3 compared to β-actin as loading control. Following normalisation for loading, the fold change in AQP3 expression was calculated relative to 295 mosm/kg control in panels a, b and c, or no treatment in panel d. Compiled results from n = 4 experiments performed using three (a and b) or two (c and d) independent NHU cell lines are presented as bar charts and show the mean fold change ± SD. Significant changes relative to control are marked as * P<0.05; ** P<0.01.

In replicate experiments using independent NHU cell lines, immunoblotting confirmed the change in AQP3 expression observed in response to NaCl (Figure 2a and 2b). Mean increases of >10-fold AQP3 protein expression were found in both proliferative (Figure 2a, left panel) and differentiated (Figure 2a, right panel) NHU cell cultures exposed for 72 hours to 500 mosm/kg medium containing NaCl. Conversely, in hypo-osmotic medium (215 mosm/kg), AQP3 protein expression was down-regulated by >30% in proliferative and differentiated cultures, respectively (Figure 2a). No detectable change in AQP3 expression was apparent following treatment with other osmotically-active solutes (Figure 2b). Moreover, a concentration- (Figure 2c) and time-dependent (Figure 2d) NaCl-mediated effect was apparent, with up-regulation of AQP3 starting at 4 hours post-exposure and maximal at 48 h post-exposure to 500 mosm/kg.

**Figure 3:**
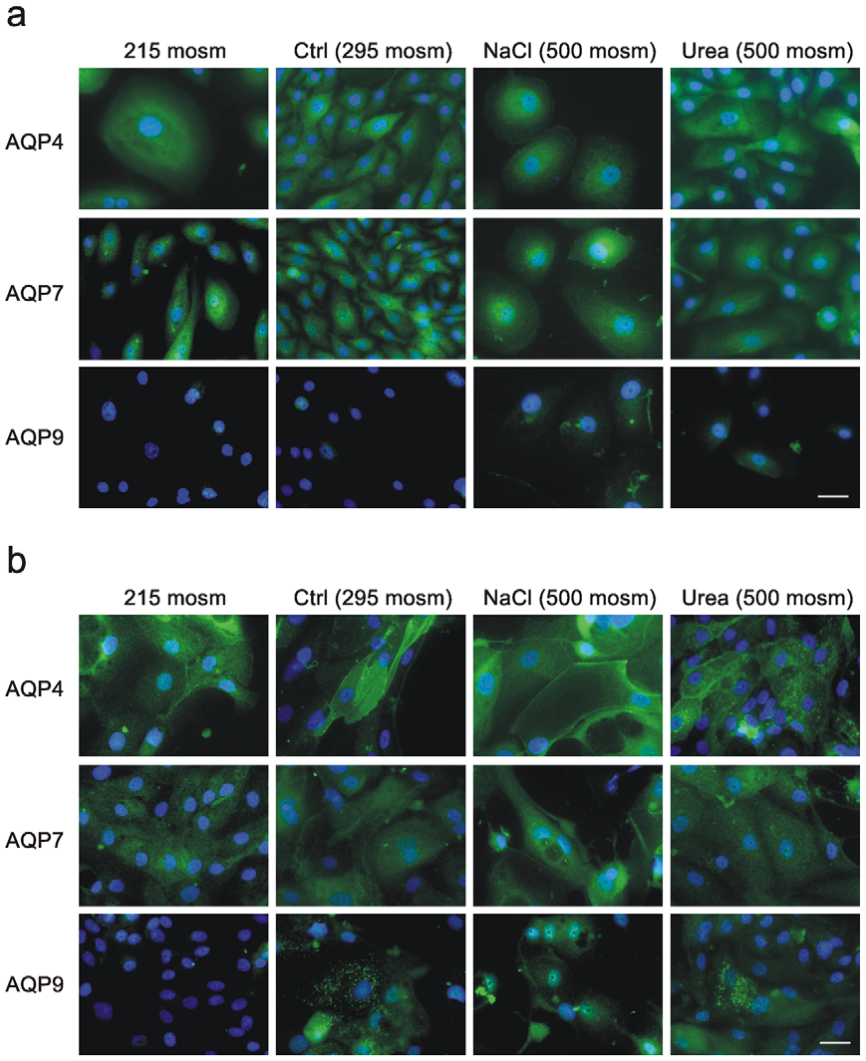
Immunolocalisation and expression of AQPs 4, 7 and 9 in response to altered medium osmolality. AQP expression and localisation was determined by immunofluorescence microscopy in proliferating NHU cultures (a) and differentiated urothelial constructs (b). AQP4 and 7 protein expression was not affected by culture osmolality. This finding was independent of the phenotype of the cells. AQP9 was expressed in the cytoplasm of differentiated cells grown under physiological conditions, in proliferative and differentiated cells exposed to hyperosmotic conditions, but was undetectable in differentiated cultures subjected to hypoosmotic culture. Scale bar: 50 µM.

Expression of AQP4 and AQP7 proteins was not affected by culture osmolality in either proliferating (Figure 3a) or differentiated (Figure 3b) urothelial cultures, as shown by immunofluorescence microscopy. AQP9 protein was present in the cytoplasm of differentiated cells grown under physiological and hyperosmotic conditions, but could not be detected when cultures were exposed to hypo-osmotic culture conditions (Figure 3b).

### Water and urea permeability of differentiated urothelial tissue constructs

**Figure 4:**
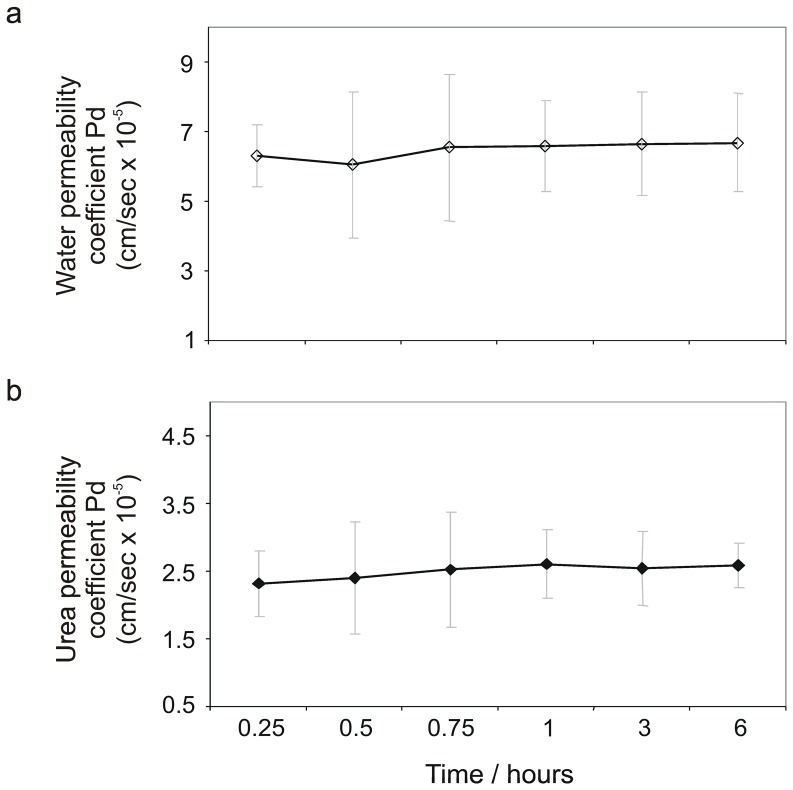
Water and urea permeability of differentiated urothelial tissue constructs. Diffusive permeability coefficients across differentiated urothelial cell cultures are shown for (a) [^3^H]-water and (b) [^14^C]-urea. Results were compiled from a total of 10 urothelial constructs from five independent cell lines. Data shown as mean ± SD.

The permeability properties of 10 differentiated urothelial constructs from five independent NHU cell lines of bladder and ureteric origin were determined by TER measurements and permeability studies using radioactively-labelled water and urea (Figure 4). The mean TER was 3863 (±772) Ω.cm^2^, indicating a ‘tight’ urothelium, whereas parallel cultures maintained in a non-differentiated state in control conditions (295 mosm/kg) had a mean TER of 24 (±7) Ω.cm^2^. Consistent with a barrier function, differentiated urothelial constructs exhibited significantly lower water and urea permeabilities than urothelial cultures maintained in a non-differentiated state (Table 1). In a subset of experiments, the osmolality of the medium was increased by adding NaCl or urea to one of the hemi-chambers. The mean osmotic water and urea permeability coefficients in the presence of a transepithelial osmotic gradient of 100 mosm/kg were 2.1-fold and 1.9-fold higher for ^3^H-water and ^14^C-urea respectively than the corresponding permeability coefficients in the absence of an osmotic gradient (based on six independent cell lines; Table 1).

**Table 1:** TER and water and urea permeability coefficients of non-differentiated monolayer cultures and differentiated urothelial constructs established on Snapwell™ membranes.

	Culture state	Non-differentiated	Differentiated
	**TER (Ω.cm^2^)**	24 (±7)	3862 (±772)
**Diffusive permeability (P_D_)**×10^−5^ cm/s (1)	[^3^H] water(2)	15.6 (±0.19)	6.33 (±0.29)
	[^14^C] urea(3)	7.2 (±0.36)	2.45 (±0.13)
**Osmotic permeability (P_f_)**×10^−5^ cm/s	[^3^H] water	not assessed	13.3 (±0.31)
	[^14^C] urea	not assessed	4.6 (±0.19)
**Number of measurements**		4	16
**Number of independent cell lines**		1	5

Results are displayed as mean ± s.d.

(1) P_D_ urothelium after correction for the P_D_ of the Snapwell™ membrane.

(2) P_D_ significantly different for cultures maintained in non-differentiated versus differentiated states (p<0.0001; unpaired 2-tailed t test).

(3) P_D_ significantly different for cultures maintained in non-differentiated versus differentiated states (p<0.0001; unpaired 2-tailed t test with Welch correction).

**Figure 5:**
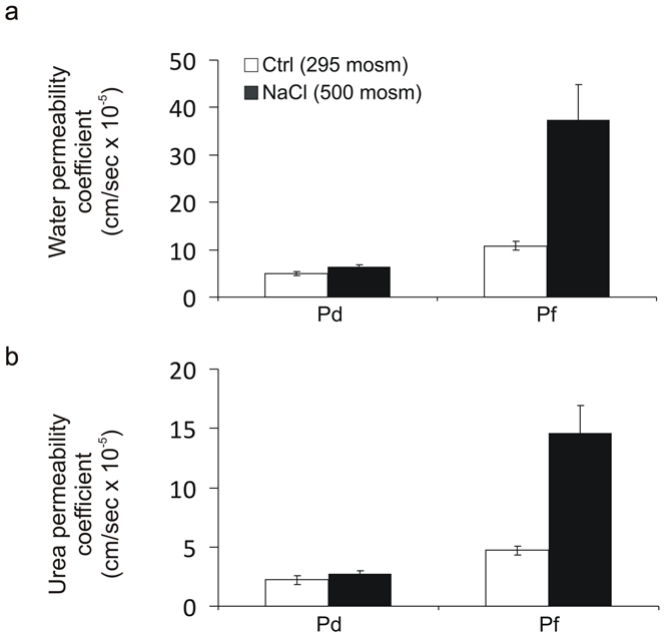
Effect of adaptation to hyperosmotic NaCl conditions on transurothelial permeability to water and urea. Differentiated NHU constructs were maintained in normal medium (295 mosm/kg) or preconditioned for 48 hours in medium adjusted to 500 mosm/kg with NaCl. Immediately prior to permeability studies, the medium was replaced with normal medium. Urothelial constructs were investigated for permeability to water (a) and urea (b) in an osmotic equilibrium (to investigate diffusive permeability: Pd) or in the presence of a 100 mosm/kg transepithelial osmotic gradient (to determine permeability flux: Pf). Pd and Pf were determined by measuring radio-isotopic fluxes from the apical compartment across the tissue to the basal compartment. Each bar shows the mean ± SD for n = 4. Adaptation to hyperosmotic conditions resulted in a significant increase in water (P<0.001) and urea (P<0.001) flux across the urothelium in the presence of an osmotic gradient, but did not affect the diffusive permeability of either (P>0.05).

Differentiated urothelial constructs preconditioned by exposure to 500 mosm NaCl-supplemented medium for 48 hours exhibited no significant change in diffusive permeability coefficient (Pd) relative to control. By contrast, a significantly higher flux (Pf) of water (3.6-fold increase) and urea (3.1-fold increase) was seen across preconditioned urothelial constructs placed in an osmotic gradient compared to (control) constructs that had not been preconditioned (Figure 5a and 5b).

### Effect of HgCl_2_ on the permeability of differentiated urothelial constructs to water and urea

NHU cell cultures were exposed to increasing concentrations of HgCl_2_, a non-selective AQP inhibitor. Cells tolerated HgCl_2_ concentrations of up to 300 µM without morphological alterations or signs of cell death; at concentrations of 500 µM and above, HgCl_2_ had a toxic effect (not shown). HgCl_2_ was added to the basal compartment of the Snapwell™ support at final concentrations of 10, 100 and 300 µM. Isotope fluxes, permeability coefficients and tracer concentration gradients were calculated from the corresponding counts.

**Figure 6:**
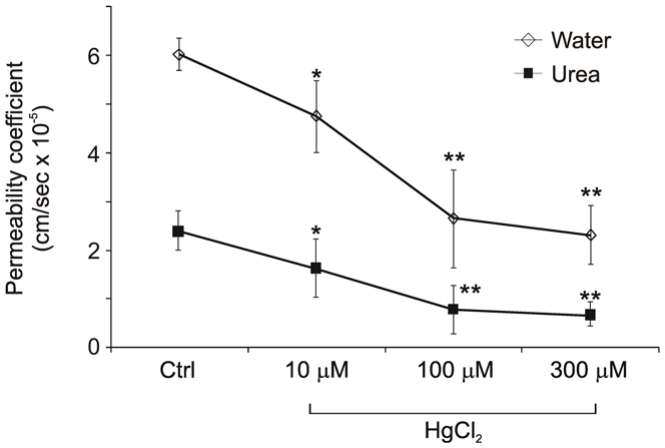
The effect of mercuric chloride on the permeability of differentiated urothelial constructs. Water and urea permeability coefficients of differentiated urothelial constructs are shown 15 minutes after application of HgCl_2_. Results are compiled from 8 independent urothelial constructs. Exposure to HgCl_2_ significantly reduced transurothelial permeability to water and urea (* P<0.05; ** P<0.01). The water permeability coefficient P_D_ diminished by 2.9 fold and the urea permeability coefficient P_D_ diminished by 3.6 fold following exposure to 300 µM HgCl_2_.

HgCl_2_ invoked a decreased flux of both isotopes and in the water and urea permeability coefficients in 8/8 differentiated urothelial constructs from two independent NHU cell lines. The decrease in water and urea permeability coefficients was apparent within 15 minutes of addition of HgCl_2_ and was HgCl_2_ concentration-dependent (Figure 6).

## Discussion

It is generally assumed that the kidney is solely responsible for the composition and final concentration of the urine. However, this assumption has been challenged by the findings of a series of in vivo studies which suggest that urine may be modified by the urinary tract. Significant net transport (both lumen to blood and blood to lumen) of urinary constituents, usually along their respective blood/urine gradients, has been demonstrated in several animal species [10], [11], [12], [13], [14]. Alongside water and urea transport, it has been shown that urothelium is capable of active transepithelial ion transport. Rabbit and guinea pig bladder urothelia have been found to contain an aldosterone-stimulated, amiloride-inhibited sodium transporter [15], [16], which is known to be responsible for salt and fluid transport across epithelia of many tissues [17]. Furthermore, molecular evidence has been provided that mammalian urothelium expresses potassium and chloride channels [18] as well as Na^+^/H^+^ and Cl_2_/HCO_3_^2−^ exchangers, which are presumed to play a role in cell volume recovery during an increase in serosal osmolality [19]. However, despite the available evidence that urinary constituents can traverse mammalian urothelia along various pathways, few studies have focused on transport mechanisms in human urothelium.

Our previous observation that AQPs are expressed by human urothelium [5] drove the present study to address the potential physiological significance of AQP expression in functional assays. The phenomenon of hypertonic induction of AQP expression has been demonstrated in several cell systems [20], [21], [22] and our finding that expression of AQP3 protein is highly responsive to NaCl concentration indicates a mechanism for regulating urothelial cell osmolality and volume. Although an in vitro study, our results have implications that extend to a role for urothelium in modulating body salt and water homeostasis, as differentiated urothelial cell constructs adapted to conditions of hyperosmotic NaCl showed significant increases in osmotic permeability, but alongside a minimal change in diffusive permeability. As AQPs are known to facilitate transepithelial water and solute transport in the presence of a pressure or osmotic gradient (“facilitated diffusion”), our results suggest that osmotic permeability coefficient (Pf) may be a more relevant measure than diffusive permeability (Pd) to reflect AQP function.

Modification of AQP expression ostensibly by osmotic challenge, as demonstrated here for the first time in human urothelium, is compatible with Spector and colleagues' findings in the rat, where dehydration led to significant up-regulation of urothelial AQPs 2 and 3 [8]. The authors suggested that AQPs may be involved in water and urea transport across the urothelium and thus in body fluid homeostasis, at least in the rat [7], [8]. Importantly, our study study extends the relevance of these observations to man.

The urothelium is exposed to extreme osmotic conditions, with urine osmolality ranging from 50 to 1300 mosm/kg, depending on the body's hydration status. Although the asymmetric unit membrane and the tight junctional complexes of the superficial urothelial layer provide a highly efficient urinary barrier, urothelial cells are likely to be rendered intermittently hypertonic as a result of the finite permeability of the barrier [23], particularly when perturbed (e.g. by infection). Thus, the ability of urothelial cells to respond to hypertonicity affords a homeostatic mechanism for regulating individual cell osmolality and volume, although further investigations determining changes to cell size in the face of osmotic challenges would be required to establish a role of AQPs in cell volume regulation.

Our finding of a low, finite permeability of cultured urothelial tissue to water and urea is suggestive of a urine-modifying role for human urothelium and is supported by independent investigations. Cahill and associates found significant differences in pH, osmolality, sodium and potassium concentrations between urine from bladder and renal pelvis [6]. They concluded that modification of urine composition in the urinary tract supported the concept of a “dynamic urothelium” and proposed that urothelial-urinary interactions and urinalysis needed reappraisal, particularly in relation to urinary stone formation and sensory bladder function [6]. Although no mechanism has yet been established, other authors have since confirmed a urine-modifying role for the urinary tract [24], [25], [26]. Also supportive of a urine-modifying role are reports of urea transporter B (UT-B) gene expression by mammalian urothelium [27], [28]. Based on its localisation and functional properties, it is conceivable that AQP3 (and potentially AQP9) may act in concert with UT-B to facilitate urea transport across the basal layers of the urothelium to the underlying capillary network of the submucosa. Spector and associates concluded that urothelial absorption of urea may become relevant in obstructive uropathy, states of dehydration and azotemia, respectively; conditions in which elevation of urea is out of proportion to the reduction in glomerular filtration rate (GFR) and the rise in serum creatinine. They hypothesized that assessment of the GFR from urea and creatinine in voided urine might underestimate renal clearance values because of reabsorption of both substances across the urothelium [27].

We have previously demonstrated the expression of AQPs 3, 4, 7 and 9 in human urothelium [5]. Our present study provides presumptive evidence that AQP3 and AQP9 are modulated by osmolality in human urothelium. In the case of AQP3, this response appears to be specific to NaCl concentration, rather than a generic response to osmotic stress, as no effect was induced by other osmotically-active solutes. By contrast, AQP9 expression was modified by exposure to NaCl or urea. Further work will be required to identify the mechanisms of AQP regulation, such as described for transcriptional regulation of AQP1 in the kidney medulla under hypertonic conditions [29].

The production of differentiated human urothelium in vitro forms the basis of a tractable experimental system for examining physiological responses to osmotic and other changes in urine composition. In particular, the model offers the advantage of a polarised system with access to basal and superficial urothelial compartments for interrogating urinary versus systemic changes. Our study provides prima facie evidence that expression of AQPs is modified in human urothelium by changes in extracellular NaCl concentration.

From this study it is tempting to speculate that AQPs and in particular AQP3 respond to urine hypertonicity and mediate facilitated diffusion of water and urea across the urothelium. However, the significance of any contribution from the urothelium to bulk water transport in situ remains to be confirmed, as caution is required in translating directly from in vitro to in situ, where other factors such as intravesical pressure, urinary composition and urothelial surface area may be significant. Despite expressing many characteristics of native urothelium, the differentiated urothelium produced in the laboratory is an approximation of urothelium in vivo. A further limitation concerns the use of mercuric chloride which, although used widely to assess aquaporin activity in plants and animals due to its high affinity for aquaporin inhibition, can at best be regarded as a non-specific AQP inhibitor. Future studies using knockdown strategies will enable the role of a specific AQP isoforms expressed by the urothelium to be elucidated. Other technical considerations in acquiring the data, such as the complex role of the “unstirred layer” in the assessment of transurothelial water and urea fluxes also limit direct extrapolation of the measurements. Finally, the osmotic permeability of untreated cultured urothelium is markedly lower than that reported for intact renal tubular epithelium (mediated via AQP1) and distal collecting ducts (AQP2), airways and lung (AQPs 3–5) [30], [31], suggesting that urothelium, is likely to play only an accessory or modulatory role. Nevertheless, the finite permeability of cultured human urothelium to water and urea, which appears to be attenuated upon functional AQP inhibition, opens up the potential for future investigations into the biological and pathophysiological significance of AQP expression by human urothelium in vivo.

When considered in toto with the emerging evidence from other studies, our results support an active role for human urothelium in sensing and responding to hypertonic salt concentrations through alterations in AQP protein expression providing a potential molecular basis for transurothelial water and urea transport. The existence and contribution of such a mechanism to normal urinary physiology has yet to be realised, but may be of particular significance in renal failure, obstructive uropathy and in disorders where the urothelial barrier is compromised, such as dysfunctional bladder syndromes and infectious or interstitial cystitis.

## Methods

### Isolation and culture of human urothelium

Collection of surgical specimens of normal human urothelium had Research Ethics Committee approval (Leeds (East) REC) and full informed written patient consent. Samples of histologically-normal bladder, renal pelvis and ureter were obtained at surgery from adult and paediatric patients with no history of urothelial dysplasia or neoplasia. Urothelium was isolated as described elsewhere and used to establish non-transformed, finite normal human urothelial (NHU) cell lines, as previously detailed [32], [33]. Cultures were maintained in complete KSFM (Invitrogen) containing 30 ng/mL cholera toxin (Sigma Aldrich; KSFMc) and used between passages 2–5.

### Differentiated urothelial tissue constructs

NHU cells were propagated in KSFMc and preconditioned for 4 days in 5% adult bovine serum (ABS) before harvest and reseeding onto glass-slides or 1.13 cm^2^ permeable Snapwell™ supports (Corning) [9]. After 24 h, the exogeneous calcium concentration was increased from 0.09 to 2 mM. Differentiated cultures were maintained in KSFMc supplemented with 5% ABS and 2 mM calcium chloride for 7 days before performing functional assays and immunohistochemistry.

### Preparation of hypo- and hyperosmolar media

Culture media were prepared by adding water, sodium chloride, glucose, mannitol or urea to KSFMc to produce culture osmolalities ranging from 215–700 mosm/kg. Osmolality was verified using a vapour pressure osmometer. NHU cell cultures were subjected to different osmotic conditions for 4 to 72 hours.

### Immunohistochemistry

Changes in AQP3, 4, 7 and 9 protein expression in response to osmotic stress were assessed immunochemically. Intact, differentiated urothelial sheets grown on Snapwell™ membranes were harvested in 2% dispase II (Sigma Aldrich), fixed in 10% formalin and processed into paraffin wax. 5 µm sections were dewaxed, antigen-retrieved by boiling for 10 min in Tris/EDTA pH 9 (AQP3) or citric acid pH 6 (AQPs 4, 7 and 9), blocked with 10% rabbit (for AQP3) or goat serum (for AQPs 4,7 and 9) to prevent non-specific binding and labelled with titrated primary and secondary antibodies (Table 2) for immunoperoxidase detection.

**Table 2:** Antibody table.

Antibody	Host	Antigen	Titrated antibody concentration (µg/ml)	Source (Cat. #)
Anti-AQP3	Goat	Human AQP3	2.0 (WB) 20 (IF) 4.0 (IHC)	Santa Cruz (sc- 9885)
Anti-AQP4	Rabbit	Human AQP4	5.0 (WB) 10 (IF) 6.6 (IHC)	Santa Cruz (sc-20812)
Anti-AQP7	Rabbit	Rat AQP7	6.6 (WB) 40 (IF) 4.0 (IHC)	Santa Cruz (sc-28625)
Anti-AQP9	Rabbit	Rat AQP9	2.0 (WB) 5.0 (IF) 2.0 (IHC)	Alpha Diagnostics(AQP91-A)

**Key**: (WB) western blotting; (IF) immunofluorescence; (IHC) immunohistochemistry.

### Immunofluorescence microscopy

Proliferative and differentiated NHU cell cultures grown on 12-well glass slides for up to 7 days were fixed in methanol∶acetone, air-dried and incubated sequentially in primary and fluorophore-conjugated secondary antibodies [5]. Immunolabelling was visualised by epifluorescence on an Olympus BX60 microscope.

### Immunoblotting

Cell lysates were prepared as previously described [34], resolved on NuPAGE™ 4–12% bis-Tris polyacrylamide gels (Invitrogen) and transferred electrophoretically onto PVDF membranes (Millipore). Membranes were incubated with titrated primary antibodies against AQPs 3, 4, 7 and 9 (Table 1) for 16 h at 4°C. Bound antibody was detected using rabbit anti-goat imunoglobulin conjugated to Alexa Fluor680 (Invitrogen) and goat anti-rabbit immunoglobulin conjugated to IRDye™ 800 (Rockland) and quantified on an Odyssey™ Infrared Imaging System (Li-Cor).

### Trans-epithelial electrical resistance (TER)

Differentiated urothelial tissue constructs grown on Snapwell™ membranes were analysed for TER using a portable EVOM™ Epithelial Volt-ohmmeter (World Precision Instruments).

### Determination of water and urea permeability

Permeability properties of urothelial tissues established on permeable Snapwell™ supports were investigated by measuring radioisotopic fluxes, in the absence or presence of mercuric chloride (HgCl_2_; non-competitive AQP inhibitor). After assessing TER, 5 µl of [^3^H]water (200 µCi/ml, Sigma) and 5 µl of [^14^C]urea (200 µCi/ml, Amersham) were dispersed in the apical hemi-chamber. During the next 60 minutes, duplicate 25 µl aliquots were taken from both apical and basal hemi-chambers at 15 minute intervals into 4 ml Ultima Gold™ XR scintillation fluid and counted on a Tri-Carb 2700TR liquid scintillation counter (Packard). After sampling, the aliquoted volume was replaced with fresh medium and the TER was checked to confirm that the urothelial culture had not been physically disturbed. Disintegrations per minute (DPM) were used to calculate a) the concentration gradient (ΔC) and the flux (Φ) of the isotope tracer across the urothelium and Snapwell™ membrane and b) the water and urea permeability coefficients (P_D_) for each sampling time point.

The measured water and urea permeabilities (P_D_) were calculated for each sampling time point using the formula PD = Φ/[A]×[ΔC] (unit: cm/sec), as described [5].

Φ: flux of the isotope tracer across the urothelium and Snapwell membrane per sec and cm^2^ (unit: µmol/sec.cm^2^).

A: area of the Snapwell membrane (1.13 cm^2^)

ΔC: concentration gradient of the isotope across the urothelium and membrane (unit: µmol/cm^3^ = µmol/ml).

[^14^C]urea and [^3^H]water fluxes (Φ) were calculated using the formula:

Tracer flux = Increase of tracer in basal hemi-chamber (µmol)/Time between samples (s)×area (cm^2^).

The increase in concentration (ΔC) of [^14^C]urea and [^3^H]water in the basal hemi-chamber was calculated from the sample DPM (where 2.22×10^6^ DPM = 1 µCi) and the specific activity of individual isotopes at the different time points (specific activity for [^14^C]Urea: 7.8 µCi/µmol and for [^3^H]Water: 0.022 µCi/µmol).

The measured permeabilities for each individual urothelial cell culture were corrected for the presence of the Snapwell™ membrane using the formula:
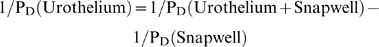



### Statistics

Experimental data were analysed using a one-way analysis of variance (ANOVA) and where significant (P<0.05), the Dunnett's multiple comparison post-test was applied to compare individual versus control datasets. Results are reported as significant where P<0.05 (*) or P<0.01 (**). Statistical tests were applied using Instat® (GraphPad Software Inc).
